# Immune evasion in cell-based immunotherapy: unraveling challenges and novel strategies

**DOI:** 10.1186/s12929-024-00998-8

**Published:** 2024-01-12

**Authors:** Yan-Ruide Li, Tyler Halladay, Lili Yang

**Affiliations:** 1 Department of Microbiology, Immunology and Molecular Genetics, University of California, Los Angeles, Los Angeles, CA 90095 USA; 2 Eli and Edythe Broad Center of Regenerative Medicine and Stem Cell Research, University of California, Los Angeles, Los Angeles, CA 90095 USA; 3 Jonsson Comprehensive Cancer Center, David Geffen School of Medicine, University of California, Los Angeles, Los Angeles, CA 90095 USA; 4 Molecular Biology Institute, University of California, Los Angeles, Los Angeles, CA 90095 USA

**Keywords:** Immune evasion, Cell-based immunotherapies (CBIs), Chimeric antigen receptor (CAR), CAR-engineered T (CAR-T) cell therapy, Tumor microenvironment (TME), Immune checkpoint proteins, Tumor heterogeneity

## Abstract

Cell-based immunotherapies (CBIs), notably exemplified by chimeric antigen receptor (CAR)-engineered T (CAR-T) cell therapy, have emerged as groundbreaking approaches for cancer therapy. Nevertheless, akin to various other therapeutic modalities, tumor cells employ counterstrategies to manifest immune evasion, thereby circumventing the impact of CBIs. This phenomenon is facilitated by an intricately immunosuppression entrenched within the tumor microenvironment (TME). Principal mechanisms underpinning tumor immune evasion from CBIs encompass loss of antigens, downregulation of antigen presentation, activation of immune checkpoint pathways, initiation of anti-apoptotic cascades, and induction of immune dysfunction and exhaustion. In this review, we delve into the intrinsic mechanisms underlying the capacity of tumor cells to resist CBIs and proffer prospective stratagems to navigate around these challenges.

## Introduction

Cell-based immunotherapies (CBIs) represent pioneering paradigms in oncological intervention, harnessing the potential of the immune system to combat malignancies [1–4]. These groundbreaking therapeutic modalities encompass the ex vivo manipulation or genetic engineering of a patient's immune cells, including T cells and natural killer (NK) cells, followed by their infusion back into the host with augmented capabilities to selectively target and eliminate cancerous cells. Two exemplary instances of cell-based immunotherapies are chimeric antigen receptor (CAR)-engineered T (CAR-T) cell therapy and CAR-engineered NK (CAR-NK) cell therapy, both of which have exhibited remarkable efficacy in addressing previously recalcitrant cancer types [5–8]. By endowing immune cells with the capacity to recognize and engage tumor-specific antigens while concurrently augmenting their cytotoxic potential, CBIs offer a highly promising avenue to surmount the limitations of conventional oncological treatments, thereby heralding an era of personalized and precision medicine. Notwithstanding their transformative potential, current CBIs are not devoid of limitations, including the propensity for off-target effects, restricted effectiveness against solid tumors, immunosuppressive influences, and the variable nature of cell manufacturing processes [1].

Immune evasion on tumor cells subsequent to CBIs poses a significant impediment to the effectiveness of CBIs [9]. This phenomenon involves the tumor cells employing strategies to elude or counteract the immune responses elicited by therapeutic manipulation of immune cells. A prevalent mechanism of immune evasion entails modifying the expression of antigens targeted by the therapeutic immune cells, thereby diminishing the tumor cells’ visibility to the immune system [9]. Furthermore, tumors may heighten the presence of inhibitory molecules, such as immune checkpoint proteins (e.g., PD-L1), which curtail immune cell activation and attenuate the anti-tumor immune reaction [10]. Additionally, the tumor microenvironment (TME) may adopt an immunosuppressive milieu, enabling tumor cells to evade immune monitoring [11]. These tumor cells might also release factors that foster immune tolerance, further undermining the potency of CBIs. Consequently, comprehending and circumventing these immune evasion tactics are imperative to optimize the outcomes of CBIs, thereby fostering enduring and efficacious anti-tumor responses.

In this review, we systematically examine the diverse array of immune evasion mechanisms harnessed by tumor cells. We particularly emphasize pivotal elements encompassing the modulation of the TME, the curtailment of tumor antigen expression, the amplification of inhibitory molecular signals, and the induction of checkpoint pathways. Furthermore, we delve into the existing methodologies aimed at surmounting immune evasion within the context of CBIs. We expound upon the utilization of amalgamative therapeutic approaches, checkpoint blockade inhibitors, and genetic engineering strategies to bolster the endurance and functionality of CBIs. Additionally, we highlight the intricacies associated with these strategies and their potential implications for clinical outcomes.

## Immune evasion mechanisms

**Fig. 1:**
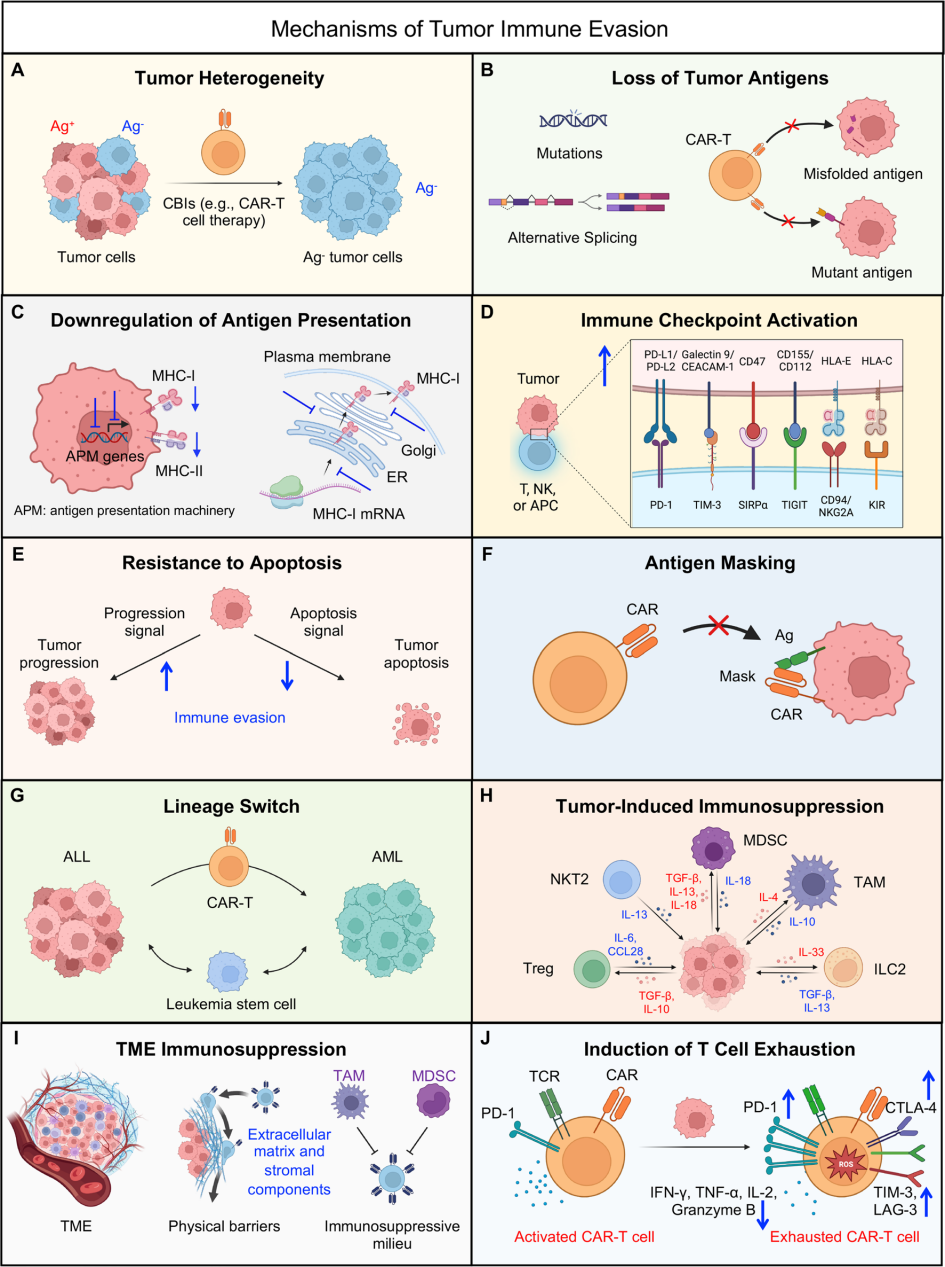
Mechanisms of tumor immune evasion. Tumor cells employ a diverse array of immune evasion mechanisms that curtail the effectiveness of cell-based immunotherapies, such as CAR-T cell therapies. These multifaceted strategies encompass tumor heterogeneity (**A**), tumor antigen loss (**B**), antigen presentation downregulation (**C**), immune checkpoint activation (**D**), apoptosis resistance (**E**), antigen masking (**F**), tumor lineage switch (**G**), tumor-induced immunosuppression (**H**), tumor microenvironment (TME) immunosuppression (**I**), and induction of T cell exhaustion (**J**)

Tumor cells frequently deploy a multitude of mechanisms to elude immune surveillance, thereby enabling their evasion from detection and elimination by the host's immune response and various CBIs. These intricate strategies play a pivotal role in fostering the unbridled proliferation and metastasis of cancer cells. Here we delve into a selection of principal immune evasion mechanisms harnessed by tumor cells (Fig. 1).

### Tumor heterogeneity

Tumor heterogeneity encompasses the phenomenon wherein distinct tumor cells exhibit varying morphological and phenotypic attributes, spanning cellular morphology, gene expression, metabolic patterns, motility, proliferation rates, and potential for metastasis [12] (Fig. 1A). Within the bulk tumor mass, a diverse assemblage of cells harboring disparate molecular imprints coexists, displaying varying degrees of responsiveness to therapeutic interventions. This heterogeneity serves as a wellspring for the emergence of resistance mechanisms [12]. Prior to any therapeutic intervention, the presence of pre-existing antigen-negative tumor cell populations within this heterogeneity can potentially underlie resistance to CBIs. To illustrate, while CD19 stands as a widely employed target for CAR-T cell therapy in the context of treating acute lymphoblastic leukemia (ALL), it is noteworthy that not all B cell malignancies express CD19 uniformly. Specifically, approximately 80% of ALL cases exhibit CD19 expression, with the percentages rising to 88% for B cell lymphomas and 100% for B cell leukemias [13]. In the realm of clinical investigations, instances have arisen wherein pre-existing CD19-negative subclones were discerned in a patient afflicted with B-ALL [14]. This pre-existing heterogeneity contributed to a scenario wherein CD19-negative relapse ensued subsequent to CAR-T cell therapy [14].

### Loss of tumor antigens by mutations and alternative splicing

In the realm of B cell malignancies, a notable phenomenon emerges wherein the loss of CD19, a pivotal cell surface antigen, has been documented in a substantial subset of patients afflicted with B cell ALL following treatment involving diverse CD19-targeting CAR-T cell therapies [15]. Similarly, within the domain of multiple myeloma (MM), a significant contingent of patients exhibited a discernible downregulation of B cell maturation antigen (BCMA) subsequent to therapeutic interventions centered on BCMA-targeting CAR-T strategies [16, 17]. These observations underscore the role of antigenic loss as a significant impediment contributing to tumor relapse (Fig. 1B).

Mechanistically, such antigenic loss has been attributed to various factors, including point mutations and alternative splicing events. Exemplifying the case of CD19-positive ALL, instances have arisen wherein point mutations within the CD19 gene have led to the production of a truncated protein, characterized by either a nonfunctional or entirely absent transmembrane domain [15, 18]. Furthermore, alternative splicing events have engendered CD19 variants marked by the absence of the extracellular epitope crucial for recognition by CAR T cells, or alternatively, have culminated in the omission of the transmembrane domain, thereby preventing the expression of CD19 on the cell surface [19–21]. This multifaceted landscape of antigenic alterations underscores the intricate mechanisms by which tumor cells can evade CAR-T cell recognition and subsequent targeting.

### Downregulation of antigen presentation

Tumor cells possess the capacity to diminish antigen presentation, thereby rendering themselves inconspicuous and compromising the ability of immune cells, particularly T cells, to identify and engage tumor-specific antigens [22] (Fig. 1C). This phenomenon can be attributed to several mechanisms. Firstly, tumor cells can induce the downregulation or acquisition of mutations in major histocompatibility complex (MHC) genes, resulting in decreased or absent expression of MHC molecules [23–26]. Secondly, perturbations in the process of loading tumor antigens onto MHC molecules can occur, encompassing alterations in the immunoproteasome activity (resulting in peptide deficiency), impairment of peptide entry into the endoplasmic reticulum through transporter associated with antigen processing (TAP), and perturbations involving chaperone proteins [22, 27]. These alterations collectively culminate in the absence of accurate peptide loading onto MHC molecules. Furthermore, effective MHC signaling to T cells can be compromised by the reduction of costimulatory molecules such as CD80 and CD86 [28, 29].

It is noteworthy, however, that the deficiency in T cell cytotoxicity due to MHC-I loss can be offset to some extent by the activation of NK cells, triggered by the 'missing-self' theory in the absence of MHC-I recognition [30]. In a counter-response, tumor cells downregulate the expression of NKG2D ligands to evade the cytotoxicity mediated by NK cells [31]. This intricate interplay underscores the dynamic strategies employed by tumor cells to subvert immune surveillance and highlights the complex balance between immune activation and evasion in the tumor microenvironment.

A disruption in the maturation and trafficking process of CD19 has been identified as a contributory factor in conferring resistance to therapies targeting CD19. CD81, an integral chaperone protein, plays a pivotal role in governing the maturation and transit of CD19 protein from the Golgi apparatus to the cellular surface [32]. Notably, in a specific patient context, it was discerned that post-transcriptional regulatory mechanisms were accountable for the loss of CD81. This consequential loss of CD81 function impeded the orderly processing and maturation of CD19 within the Golgi apparatus, thereby undermining the proper localization of CD19 to the cell surface [32].

### Immune checkpoint activation

Tumor cells adeptly exploit immune checkpoint pathways as a strategic maneuver to subdue immune responses. Through interactions with these checkpoint molecules, tumor cells can effectively quell T cell activity and attenuate the immune reaction [33] (Fig. 1D). Among the most extensively investigated and recognized inhibitory checkpoint pathways are cytotoxic T lymphocyte-associated molecule-4 (CTLA-4), programmed cell death receptor-1 (PD-1), and programmed cell death ligand-1 (PD-L1) [33]. Innovative therapeutic approaches have been adeptly developed to target these pivotal molecules and their associated pathways.

CTLA-4 exhibits constitutive expression in regulatory T cells and is induced subsequent to T cell activation via CD28 and TCR signaling [34, 35]. CTLA-4, along with its homologous counterpart CD28, is expressed by both CD4^+^ and CD8^+^ T cells, exerting opposing regulatory functions in the context of T cell activation [34]. CD28 engages with the CD80 dimer with relatively high affinity and the CD86 monomer with lower affinity, thereby facilitating T cell costimulation in tandem with TCR signals [36, 37]. In contrast, interactions of these ligands with CTLA-4 serve to inhibit T cell responses, although the precise underlying mechanisms remain incompletely understood. The CD28/CTLA-4 pathway has garnered substantial interest in therapeutic contexts, where antibodies and fusion proteins are emerging as viable modalities [38]. The regulatory role of CTLA-4 in constraining immune responses to self-tissues renders augmentation of this pathway a potential strategy for autoimmunity treatment. Conversely, suppression of CTLA-4 holds promise for stimulating anti-self responses against tumors [38]. The milestone achievement of inducing immunological rejection of cancer through anti-CTLA-4 antibodies has catalyzed significant momentum in this field, opening avenues for innovative therapeutic approaches.

The inhibitory effects of PD-1/PD-L1 engagement on activated T cells are firmly established in both physiological and pathological contexts [39]. In the realm of cancer therapeutics, the utilization of checkpoint blockade, particularly through anti-PD-1 and anti-PD-L1 antibodies, is progressively becoming a standard treatment for an expanding spectrum of tumors [40]. PD-1 expression is swiftly induced on T cells subsequent to TCR-mediated activation, with a subsequent decline upon antigen clearance. In chronic disease settings, however, PD-1 expression persists on antigen-specific T cells and is correlated with a progressive loss of T cell functions [41]. For transient PD-1 expression on activated T cells, TCR-mediated stimulation induces dephosphorylation of NFAT, facilitating its translocation into the nucleus. Upon association with the AP-1 complex, activated through CD28 signaling, this complex drives the expression of effector genes and PD-1 [41]. Conversely, for sustained PD-1 expression on exhausted T cells, PD-L1 ligation induced by IFN-γ in the microenvironment triggers the PD-1 pathway, inhibiting TCR and CD28 signaling and subsequently reducing AP-1 activation [41]. Once translocated into the nucleus, NFAT drives exhaustion genes and maintains constant PD-1 expression, facilitated by a constitutively demethylated *PDCD1* promoter [41]. Given the inhibitory impact of the PD-1/PD-L1 axis on T cell activation and the antitumor response, checkpoint blockade therapy involving anti-PD-1 and anti-PD-L1 antibodies has found widespread application in the treatment of various cancers, especially solid tumors [39]. These therapeutic approaches have demonstrated a reinvigoration of pre-existing tumor-specific T cells, augmentation in the infiltration of CD8^+^ T cells, and ultimately the induction of tumor suppression [39, 41, 42].

Moreover, T cell immunoglobulin mucin-3 (TIM-3), initially identified in Th1 cells, assumes a pivotal role in suppressing Th1 responses and the expression of cytokines such as TNF and INF-γ [43, 44]. Elevated TIM-3 expression is concomitant with the dampening of T cell responses and the induction of T cell exhaustion [43]. This gradual loss of T cell function occurs in a hierarchical manner, particularly evident during persistent viral infections and tumorigenesis [43]. TIM-3 functions as a critical checkpoint in tumor immunity, exerting regulatory control over T cell exhaustion within tumor infiltrating leukocytes (TILs) from both human and mouse tumors [45, 46]. Notably, the expression of TIM-3 on CD8^+^ TILs is closely associated with PD-1 expression [46]. Furthermore, TIM-3 has been observed on tumor cells, where it modulates the expansion of myeloid-derived suppressor cells (MDSCs), leading to the subsequent suppression of T cell function and a resultant attenuation of immune reactivity [43]. Notably, research underscores the significance of B7-H4, prominently expressed in tumors, which exerts a dual role by dampening T cell-mediated immune responses while concurrently fostering tumor tumorigenicity [47]. Tumor cells can further evade immune surveillance by upregulating the CD47 “don’t eat me” signal, a defensive mechanism that discourages phagocytosis. Inhibition of CD47 emerges as a prospective strategy to enhance the elimination of tumor cells across diverse cancer types [48].

### Resistance to apoptosis

Tumor cells have the capacity to develop intricate strategies to counteract the cytotoxic impacts induced by introduced T cells, which encompass augmenting levels of anti-apoptotic proteins or modifying their apoptotic pathways [49] (Fig. 1E). This adaptive response empowers tumor cells to evade annihilation by immune cells, which typically eradicate aberrantly proliferating cells. The phenomenon of apoptosis resistance in cancer cells can be orchestrated through various mechanisms, such as the amplification of oncogenic signals or the attenuation of tumor suppressor gene expression [49]. In the context of human malignancies, the heightened expression of anti-apoptotic proteins such as Bcl-2, Bcl-xL, or Mcl-1 is a frequently observed occurrence [50]. This phenomenon is closely linked to the perpetuation and advancement of the disease, engendering resistance to CBIs and correlating with unfavorable clinical outcomes.

### Antigen masking

Tumor cells employ a strategic defense by enveloping their surface with molecules that hinder immune cell recognition and binding to tumor antigens (Fig. 1F). A notable clinical case involving the treatment of B-ALL through CD19-targeting CAR-T cell therapy underscores this phenomenon. In this instance, tumor relapse ensued due to the camouflage of the targeted antigen. Unexpected viral transduction of a leukemic cell during cytapheresis resulted in the coating of the B-ALL leukemia cell surface with the CAR construct itself [51]. This masking of the antigen by the CAR configuration led to an intriguing auto-recognition scenario, wherein the tumor-expressed CAR rendered the antigen effectively imperceptible to the CAR-T cells, thereby impairing their recognition and subsequent targeting efficacy [51, 52].

### Lineage switch

Lineage switch denotes a scenario in which a patient experiences a relapse marked by a genetically related yet distinct malignant phenotype, frequently observed in cases of acute myeloid leukemia (AML) (Fig. 1G). This phenomenon predominantly manifests in individuals carrying MLL rearrangements, particularly infants diagnosed with B-ALL. Lineage switch emerges when the leukemic cellular profile undergoes a transition from lymphoid to myeloid lineage [53, 54]. This phenotypic evolution is characterized by the loss of CD19 expression and the acquisition of myeloid-associated traits, emblematic of AML [55]. This transdifferentiation process is intricately linked to a profound epigenomic reprogramming that sustains tumor progression [56]. Furthermore, this remarkable phenomenon has been replicated in murine leukemia models, where preclinical studies have meticulously illustrated how CD19 CAR induction induces lineage switch within an ALL model in mice. This lineage switch is particularly dependent on the presence of the E2a:PBX transgene, akin to MLL rearrangement, which is recognized for its capacity to drive the development of either lymphoid or myeloid neoplasms [57].

### Tumor-induced immunosuppression

Beyond the occurrence of antigen loss, several additional mechanisms curtail the effective recognition of cancer cells by therapeutic entities like CAR-T cells, influenced either by tumor cells directly or by the restructuring of the microenvironment (Fig. 1H). Tumor cells exhibit the capacity to secrete factors such as transforming growth factor-beta (TGF-β) and interleukin-10 (IL-10), fostering an environment of immunosuppression [58]. This milieu encompasses an array of cytokines, chemokines, and assorted molecules that hinder the activity of immune cells, notably T cells and NK cells. TGF-β, with its pleiotropic nature, emerges as a potent immunosuppressive cytokine that curbs T-cell activation, proliferation, and differentiation [59]. Meanwhile, IL-10 prevalence within the TME compromises the functionality of dendritic cells (DCs) and safeguards tumor cells from cytotoxic T lymphocyte (CTL)-mediated cytotoxicity by downregulating TAP1 and TAP2 [60, 61]. Additional agents, including prostaglandin E2 (PGE2) [62] and sialomucins [63], have also garnered attention for their capacity to impede the function of immune cells.

### Tumor microenvironment immunosuppression

Tumors have the ability to attract immunosuppressive cells, including TAMs, MDSCs, and Tregs, to their microenvironment [64–66]. These cells actively suppress the function of immune effector cells and contribute to the establishment of an immunosuppressive milieu (Fig. 1I). Moreover, tumor cells possess the capacity to modify the extracellular matrix and surrounding stromal components, creating physical barriers that impede immune cell infiltration and compromise their functionality within the tumor milieu [67].

A noteworthy reciprocal relationship exists between cancer stem cells (CSCs) and the TME. In this dynamic interplay, the TME supports the maintenance of CSCs in a stem-like state, thereby fostering their survival, self-renewal, and resistance to therapeutic interventions through complex cellular and molecular mechanisms [68–70]. Conversely, CSCs generate factors that drive the polarization and prolonged existence of the TME in an immunosuppressed condition [71–74]. This intricate interplay between tumor cells and the TME collectively contributes to enhanced resistance of tumor cells to therapeutic interventions.

### Induction of T cell exhaustion

Tumor cells possess the capacity to induce T cell exhaustion, diminishing their functional responsiveness (Fig. 1J). This exhaustion leads to a decline in cytokine production and cytotoxic activity within T cells, ultimately compromising their efficacy in targeting tumor cells. In preclinical models, particularly within solid tumor contexts, it has been demonstrated that CAR-T cells infiltrating tumors experience rapid functional deterioration, constraining therapeutic potential. Notably, this state of hyporesponsiveness is reversible when T cells are removed from the tumor microenvironment. It is linked to the elevation of intrinsic T cell inhibitory enzymes (diacylglycerol kinase and SHP-1) and the upregulation of surface inhibitory receptors (PD1, LAG3, TIM3, and 2B4) [75].

The orchestration of T cell exhaustion involves both intrinsic and extrinsic factors. Cancer cells and stromal cells, including tumor-associated dendritic cells, regulatory T cells, TAMs, and MDSCs, exert significant extrinsic regulation on T cell exhaustion [64, 76]. Furthermore, the involvement of extrinsic cytokines such as IL-10 and TGF-β is pivotal in driving the exhaustion process within T cells. Among the intrinsic regulatory elements, inhibitory receptors such as PD-1, CTLA-4, Tim-3, BTLA, LAG-3, and TIGIT play crucial roles in shaping the trajectory of T cell exhaustion [76].

## Current strategies to overcome immune evasion

**Fig. 2:**
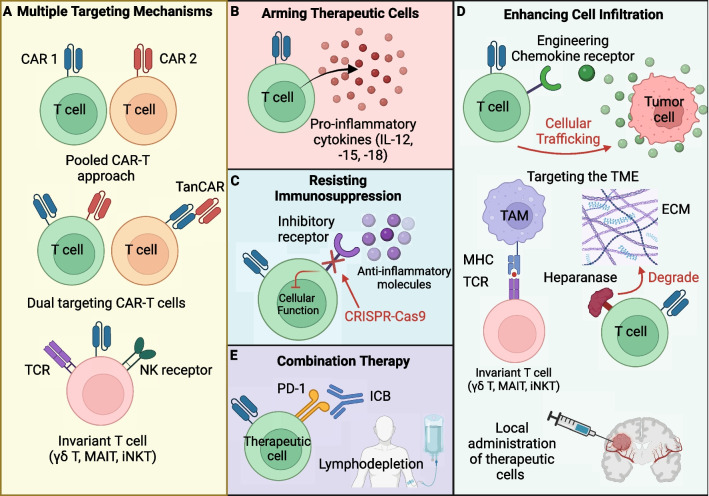
Engineering strategies to overcome immune evasion. Innovative engineering strategies have been deployed to substantially enhance the recognition of tumors, the durability of therapeutic cells, their ability to infiltrate the tumor microenvironment, and the overall effectiveness of cellular therapies. These strategies encompass a range of techniques, including the incorporation of multiple targeting mechanisms (**A**), fortifying therapeutic cells with cytokines (**B**), modifying the immunosuppressive tumor microenvironment (**C**), amplifying cell infiltration capabilities (**D**), and employing combination therapies (**E**). TanCAR, tandem CAR; iNKT, invariant natural killer T; MAIT, mucosal associated invariant T; γδ T, gamma delta T; ICB, immune checkpoint inhibitor; ECM, extracellular matrix

The mechanisms of immune evasion are a prevalent obstacle hindering the efficacy of cell-based therapies and overcoming these obstacles can greatly improve the therapeutic potential of cell-based therapies for both solid and liquid tumors. Novel engineering approaches have been used to greatly improve tumor recognition, persistence, tumor microenvironment infiltration, and overall anti-tumor efficacy of cellular therapies. Engineering approaches are summarized below (Fig. 2).

### Engineering of multiple targeting mechanisms

Antigen escape is a common barrier that significantly reduces the effectiveness of CAR-T cell therapies. This phenomenon occurs due to the decreased presence of CAR antigens, impeding the CAR-T cells' ability to identify tumor cells. A promising solution to counter this escaping mechanism involves utilizing multiple CAR-T cells called a “pooled CAR-T approach” (Fig. 2A). Each type of CAR-T cell targets a different tumor antigen, therefore reducing the likelihood of tumor cells evading recognition. For example, the IL-3 receptor α chain (CD123) is expressed in several hematological neoplasms and retains expression in B-ALL patients after CD19 targeted CAR-T cell therapy [77, 78]. Simultaneously administering CD19 CAR-T and CD123 CAR-T cells prevented CD19-loss relapse in B-ALL xenograft models and enhanced the tumor killing efficacy, demonstrating the importance of targeting multiple tumor associated antigens (TAAs) [77]. A clinical trial involving 89 patients with ALL tested the safety and efficacy of the combined infusion of CD19 CAR-T and CD22 CAR-T cells and observed a disease-negative response rate of 96.0% and 1 case of relapse due to antigen-loss, further demonstrating the impact of targeting multiple tumor antigens in order to reduce antigen-loss relapse [79].

An alternative to the “pooled CAR-T approach” that is less complex and costly to manufacture and addresses antigen escape [80], is the modification of CAR-T cells with multiple targeting mechanisms including: multiple CAR vectors, bispecific vectors that encode both CARs, or bispecific receptors called tandem CAR (TanCAR) where one CAR encodes two scFv domains for two different tumor antigens [81]. These engineering strategies empower a single CAR-T cell to engage multiple tumor antigens and enhance their capacity to combat antigen escape. For example, dual targeting CAR-T cells expressing the combined CD19 and CD123 CAR demonstrated superior in vivo activity against B-ALL compared to single and pooled combination CAR-T approaches [77]. Within a phase 1 trial in pediatric and young adult patients (n = 15) with relapsed and refractory B-ALL, CAR-T cells with a bicistronic γ-retroviral vector that encodes both CD19 and CD22 CAR has also proven efficacious resulting in a remission rate of 86% [80]. The dual targeting tanCAR proved promising within a single-arm phase 1/2a clinical trial testing the safety and efficacy of a tanCAR targeting CD19 and CD20 for 28 patients with relapsed/refractory non-hodgkin's lymphoma [82]. The study observed an overall response rate of 79% and a complete response rate of 71% [82].

In addition to the pooled CAR-T cell approach and engineering T cells with multiple CARs, using alternative cell types including NK, invariant natural killer T (iNKT), mucosal associated invariant T (MAIT), and gamma delta T (γδ T) cells provides the advantage of targeting tumor cells via multiple killing mechanisms in addition to CAR alone [82–85]. Unconventional T cells have the intrinsic advantage of having multiple killing mechanisms involving their TCR and natural killer receptors (NKRs) [82–85]. Therefore, decreasing the risk of antigen escape and tumor relapse heightens their potential for CAR engineering and their use for cell-based therapies. Overall, these strategies represent innovative approaches to address the challenge of antigen escape, enhancing the potential of CAR-T cell therapies in terms of both efficacy and durability.

### Arming therapeutic cells to enhance persistence

One approach to enhance the persistence, expansion, and long-term functionality of CAR-T cells within the hostile and immunosuppressive TME involves the genetic modification of these therapeutic cells to express proteins in addition to the CAR engineering. This strategy includes the engineering of cells to secrete inflammatory cytokines, thereby amplifying their functional capabilities and anti-tumor efficacy [86] (Fig. 2B). Arming the CAR-T cells provides the advantage of enhancing CAR-T cell expansion, persistence, survival, and anti-tumor efficacy in addition to shifting the immunosuppressive cytokine profile within the TME to an immune-activating and inflammatory state [86–89]. For instance, engineering CD19 CAR-T cells to constitutively express the pro-inflammatory cytokine IL-18 [90], has demonstrated improved CAR-T cell proliferation and anti-tumor activity compared to unarmored CD19 CAR-T cells and had adjuvant properties activating the endogenous immune system [88]. Similar approaches involving arming CD19 CAR-T cells with pro-inflammatory molecules IL-12 and IL-15 have displayed promise [87, 89]. Equipping CD19 CAR-T cells with IL-12 retained a central memory-effector phenotype with increased anti-tumor efficacy in vitro while IL-15 armed CD19 CAR-T cells had increased cell expansion, reduce cell death rate, decrease expression of PD-1 and improved anti-tumor effects in vivo compared to unarmored CD19 CAR-T cells [89]. While promising, arming CAR-T cells with IL-12 and IL-15 can potentially have toxicity issues as a phase 2 study of administering recombinant human IL-12 systemically for patients with metastatic renal cell carcinoma resulted in severe toxicities where 12 patients are hospitalized and two patients died out of the 17 patients involved [91]. Additionally, a clinical trial for the systemic administration of IL-15 to treat metastatic cancers was met with toxicity issues as well [92]. To mitigate potential toxicity issues, a more controlled approach involves cytokine secretion upon CAR antigen binding, thereby reducing toxicity caused by constitutive expression of proinflammatory molecules [93]. Overall, these studies highlight the potential of arming CAR-T cells in order to enhance their efficacy and further exploration to understand their impact on cancer therapy and understanding their limitations is warranted.

### Resisting the immunosuppressive TME

The TME exhibits high immunosuppressive qualities due to the recruitment and elevated presence of TAMs, MDSC, and Tregs and immunosuppressive molecules including IL-10 and TGF-β that suppress the function of effector cells [64–66]. Cell-based therapies face these same immunosuppressive challenges, making it crucial to discover methods to counteract this immunosuppressive for their success (Fig. 2C). One approach that has demonstrated promise is to prevent the signaling caused by immunosuppressive molecules ubiquitous within the TME. Using CRISPR-Cas9 approaches, Na Tang et al. showed that knocking out the endogenous TGF-β receptor II (TGFBR2) within CAR-T cells decreased the conversion into Treg, prevented cell exhaustion, and enhanced the anti-tumor response in in vivo xenograft models on pancreatic carcinoma [94]. A similar approach is to engineer a dominant-negative TGF-βRII (dnTGF-βRII) to decrease TGF-β availability and block its signaling within PSMA targeting CAR-T cells for the treatment of TGF-β secreting prostate cancer [95]. Doing so enhanced the PSMA-targeting T cell’s proliferation, cytokine secretion, resilience against exhaustion, longevity, and effectiveness against tumor growth in prostate cancer models [95]. Taking into consideration the benefits of creating TGF-β resistant CAR-T cells, a phase 1 clinical trial was conducted to assess the effectiveness of PSMA-targeting CAR-T cell therapy engineered with dnTGF-βRII in treating metastatic castration-resistant prostate cancer [96]. The study emphasized the feasibility of this therapy but highlights the need for further optimization for the treatment of solid tumors [96].

Analogous to TGF-β, adenosine is another immunosuppressive molecule ubiquitously produced by ectoenzymes CD73 and CD39 on tumor cell surfaces that has been shown to potently suppress T cell activity [97–99]. Of the three adenosine receptors expressed by T cells (A2AR, A2BR, and A3R) [100–102], downstream A2AR signaling is primarily responsible for suppressing cytokine production (IFN-γ, TNF-α, and IL-2), cytotoxicity, and proliferation within T cells [97]. Efforts to counteract the impact of adenosine on CAR-T cell therapy involve the deletion of the A2A receptor, a modification that renders them resistant to adenosine [98]. Using CRISPR-Cas9 to knockout the A2A receptor in Her2-targeting CAR-T cells, Giuffrida et al. showed the modification increased transcription of pro-inflammatory cytokines and improve anti-tumor efficacy in in vivo breast cancer models [98]. Similar genetic engineering approaches have been applied to mitigate the effects of PD-1 signaling [103]. Knocking out PD-1 in CAR-T cells demonstrated enhanced CAR-T cell function and clearance of PD-L1^+^ tumor xenografts in vivo [103].

In addition to resisting the immunosuppressive TME, efforts have been made to target and remodel the TME into a more inflammatory state by using innate T cells to target tumor associated macrophages [65, 104]. TAMs make up to 50% of the solid tumor mass and facilitate the exhaustion of effector T cells through production of anti-inflammatory cytokines and expression of PD-L1 [105, 106]. Li et al. have demonstrated the potential of using MAIT, iNKT, and γδ T cells to target both tumor cells and immunosuppressive TAMs via TCR dependent mechanisms [104]. Overall, these engineering strategies and use of innate T cells have demonstrated significant potential in preclinical models and present novel opportunities for genetically enhancing therapeutic cells to withstand and remodel the immunosuppressive TME.

### Enhancing therapeutic cell infiltration

Another major hurdle limiting the efficacy of cell-based therapies for the treatment of solid tumors is due to the hindered infiltration of immune cells into the TME. Tumor cells modify the extracellular matrix (ECM) and adjacent stromal composition, leading to the creation of physical barriers that decrease cellular infiltration and their functionality within the TME [67]. Additionally, tumor induced angiogenesis driven by the imbalance of pro-angiogenic factors including VEGF-A, give rise to unorganized, immature, and thin-walled vascular networks that hinder immune cell accessibility within the TME [107]. To address the challenge of limited cellular infiltration, the engineering of chemokine receptors that modulate cellular trafficking has been utilized to augment the entry of CAR-T cells into the TME (Fig. 2D). Evidently, the overexpression of CXCR2 in GPC3 CAR-T cells enhanced the in vivo trafficking, accumulation, and anti-tumor efficacy of these cells in hepatocellular carcinoma xenograft models that express high levels of CXCR2 ligands [108]. The engineering of CCR6, the receptor for CCL20, within epidermal growth factor receptor (EGFR)-targeting CAR-T cells led to improved trafficking, penetration, and clearance of solid tumors in a CCL20^+^ lung cancer xenograft mouse model [109]. Similarly, the incorporation of the CCR2b receptor, which binds to CCL2, in CAR-based therapies improved cellular trafficking in preclinical mouse models for both malignant pleural mesothelioma (MPM) and neuroblastoma. This genetic modification resulted in enhanced anti-tumor efficacy of CAR-T cells expressing the CCR2b receptor [110, 111]. These efforts highlight the impact of overexpressing the “matched” chemokine receptors that correspond to ligands naturally found in TMEs for CAR-T cell therapy in order to enhance cellular infiltration and therefore anti-tumor efficacy.

Alternative to exploiting chemotaxis dynamics to enhance cellular infiltration, equipping CAR-T cells with the capability to target and degrade components of the ECM that create physical barriers represents a promising approach to improve the efficacy of cell-based therapies (Fig. 2D). The enzyme heparanase (HPSE) degrades heparan sulphate proteoglycans which constitute the majority of the ECM [112]. The engineering of CAR-T cells to express HPSE improved their capacity to degrade ECM and subsequently improved cell infiltration and tumor eradication, therefore demonstrating the potential of targeting the barrier in treating solid tumors [112]. In a study conducted by Li et al., they revealed the potential of employing invariant T cells for the purpose of targeting immunosuppressive TAMs. This approach holds promise in reshaping the TME by reducing the presence of TAMs, which otherwise obstruct cellular infiltration [104].

Finally, numerous phase I clinical investigations have explored the outcomes of delivering CAR-T cells directly to the local and regional tumor areas, with the goal to overcome the challenge of limited cellular trafficking into the TME [113]. These studies have demonstrated that the locoregional delivery of CAR-T cells is both safe and feasible, therefore providing another way to address the obstacle of reduced cellular infiltration.

### Combination therapy

Checkpoint blockade (CPB) therapy is an effective approach used to reactivate an overwise exhausted immune cells within the TME [39, 114]. PD-1 is one of many checkpoint ligands upregulated within the TME that act to deactivate an immune response [39, 40, 114]. Signaling via PD-1 inhibits proliferation and cytokine secretion [115], facilitates Treg differentiation [116], and induces cell death [117]. In addition to tumor cells, PD-L1 is rampantly expressed on fibroblasts [118], dendritic cells, macrophages, and B and T cells [39] and their effects on diminishing CAR-T cell function is highlighted as overexpression of PD-L1 and PD-L2 on tumor cells inhibits CAR-T cell function [119]. The combined approach of PD-1 immune checkpoint blockade offers the benefit of synergizing with adoptive cell-based therapies, thereby enhancing the effector function of exhausted therapeutic cells (Fig. 2E). Pre-clinical studies evaluating the efficacy of combining anti-PD-1 blockade with Her2-targeting T cells showed significantly enhanced CAR-T cell function in two different in vivo tumor models using Her2 + sarcoma and breast cancer [120]. Li et al. reported on the use of PD-1 inhibitors in combination with CD19-directed CAR-T cell therapy for 14 patients with heavily pretreated B-ALL who initially had poor response to CD19 CAR-T therapy [121, 122]. 7 of the 14 patients maintained either partial response (PR) or complete response (CR) while 3 patients re-established B cell aplasia, an indicator of CAR-T cell function [121, 122]. The study provided evidence supporting the effective and safe use of combining checkpoint blockade with CAR-T therapy in children with relapsed B-ALL [121, 122]. Checkpoint blockade offers a means to improve CAR-T cell therapy and can prove advantageous in a clinical context that warrants further investigation in other forms of immune checkpoint blockade.

Other combination therapies that have synergistic potential with CAR-T therapy include using radiation to sensitize heterogeneous tumors to CAR-T cell killing and mitigate antigen escape [123] (Fig. 2E). Additionally, chemotherapy has also provided a potential for lymphodepleting conditioning in order to enhance CAR-T expansion, engraftment, reduce immunosuppressive cells, and increase anti-tumor efficacy [124–127]. The synergistic strategies employed in combination therapy offer substantial potential and can overcome the challenges associated with utilizing CAR-T cell therapy for solid tumors. A novel approach that is still in its infancy is the use of messenger RNA (mRNA) vaccine encoding CAR antigens (CARVac) to expand CAR-T cells against solid tumors [128]. Reinhard et al. demonstrates the potential of using a nanoparticulate RNA vaccine encoding the CAR antigen claudin 6 (CLDN6), an oncofetal cell-surface antigen suitable for CAR-T cell targeting. The CARVac strategy targets dendritic cells to express CLDN6 on the surface, subsequently activating CLDN6-CAR-T cells against solid tumors [128]. Using the mRNA vaccine approach to expand and activate CAR-T cells provides a platform to improve the engraftment of CAR-T cells and allows for therapeutic tumor control at lower CAR-T cell doses and can potentially improve CAR-T cell efficacy in a clinical setting [128]. A phase 1 dose escalation clinical trial involving 22 patients with relapsed or refractory solid tumors demonstrated the CLDN6-CAR-T plus CARVac approach was well tolerated and had promising response rates. The unconfirmed objective response rate was 33% (7/21) and the disease control rate was 67% (14 of 21) in 21 evaluable patients.

Advancements in engineering approaches are paving the way for the development of next generation CAR-T cells that can overcome many limitations associated with targeting solid tumors. Designing therapies to target multiple tumor associated antigens via engineering of dual targeting CARs or utilizing invariant T cells for CAR engineering offers the advantage of having multiple killing mechanisms that can mitigate relapse due to antigen escape and masking. Additionally, the engineering of therapeutic cells using CRISPR-Cas9 has proven effective in enhancing the infiltration, persistence, and overall tumor killing potential of therapeutic cells [129]. However, toxicity is an ever-present limitation that limits the use of cell-based therapies. Therefore, designing therapeutic cells with inducible systems or use of logic gating systems can greatly decrease negative side effects and expand its use [130]. Overall, the next generation of cell-based therapies will likely utilize many of the approaches discussed to overcome the issues of immune evasion intrinsic to treating solid tumors.

## Biomarkers for predicting immune evasion

**Fig. 3:**
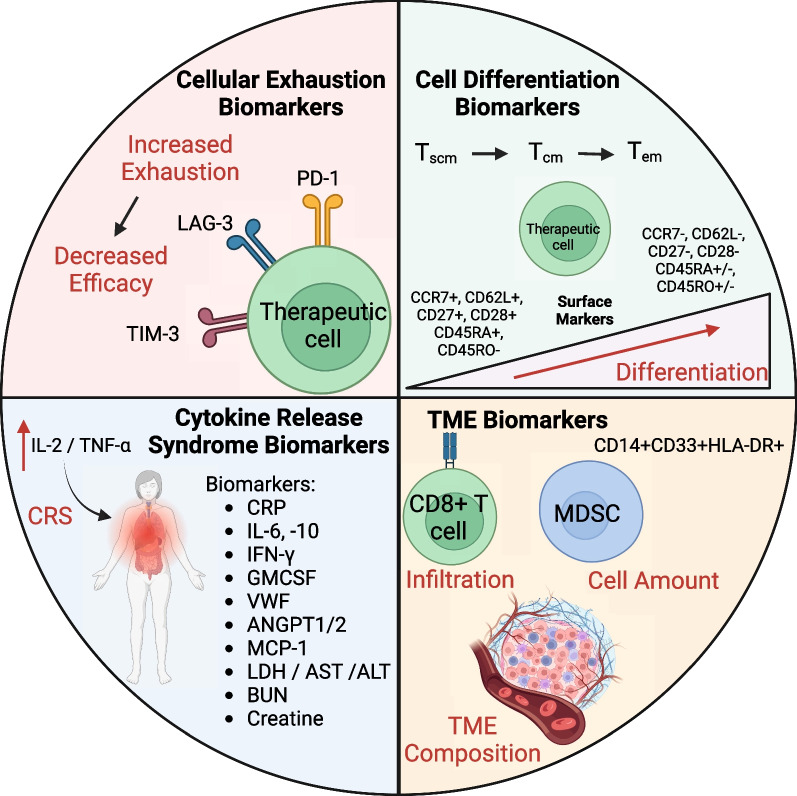
Biomarkers for cell-based immunotherapy. Biomarkers have emerged as invaluable tools in the realm of immunology and cancer therapy, playing a pivotal role in predicting several critical aspects of the immune response. Specifically, these biomarkers have found widespread application in forecasting immune cell exhaustion, a state where immune cells lose their functionality and become less effective in combating diseases. Moreover, they contribute to the anticipation of immune cell differentiation, providing insights into how immune cells transform into specialized subsets with distinct functions. Biomarkers are also instrumental in predicting the onset of cytokine release syndrome (CRS), a potentially severe immune-related side effect of certain therapies. Additionally, they aid in the assessment of the tumor microenvironment (TME), offering crucial information about the dynamic interplay between immune cells and the tumor, which is indispensable for designing personalized and effective treatment strategies

Cell based therapies are becoming increasingly prevalent in treating cancer malignancies and therefore identifying predictive biomarkers for these therapies can lead to more effective and safer treatment strategies for patients. Efforts to establish biomarkers to predict the short- and long-term effects, immune evasion, and negative side effects of cell-based therapy are ongoing and will enhance their therapeutic capacity [131, 132]. The diverse biomarkers employed to anticipate various facets of cell-based therapy are discussed below (Fig. 3).

### Immune cell exhaustion biomarkers

Exhaustion markers encompassing inhibitory checkpoint molecules define the activation and exhaustion status of CD8^+^ T cells, offering a means to predict the characteristics and effectiveness of cell-based therapies [133]. Exhausted T cells are characterized to have impaired ability to secrete IL-2, TNF, and IFN-γ and high expression of immune checkpoint molecules PD-1, TIM-3, and LAG-3 [134, 135]. Ligands for immune checkpoint molecules are ubiquitously expressed within the TME and suppress immune cell activation, proliferation, and functionality [33]. Therefore, assessing cellular exhaustion markers provide a biomarker to potentially predict the outcomes of cancer patients treated with cell-based therapies. Within a phase 1 clinical study involving 43 pediatric R/R ALL subjects, Finney et al. observed higher levels of exhaustion markers PD-1 and LAG-3 on CD8^+^ T cells apheresis starting material among the dysfunctional response group compared to functional response subjects [136]. Their study also highlighted peripheral CD8^+^ T cells expressing LAG-3^high^/TNF-α^low^ at the time of apheresis as a biomarker associated with suboptimal response to therapy [136]. Clinical analysis comparing CR to PR and NR patients with chronic lymphocytic leukemia (CLL) treated with CD19 targeting CAR-T cells observed lower percentages of CD8^+^PD-1^+^ for CR patients [137]. Further analysis uncovered that increased infusion of CD8^+^LAG-3^+^PD-1^+^ and CD8^+^TIM-3^+^PD-1^+^ CD19-targeting CAR-T cells were associated with poorer responses [137]. Overall, these clinical studies highlight the importance of considering the exhaustion status of cell-based products and their application as biomarkers to predict the efficacy of cellular therapies.

### Immune cell differentiation biomarkers

In addition to assessing the exhaustion status of therapeutic cells, distinguishing the differentiation state of therapeutic cells offers an alternative method to predict patients’ response to cell therapy. Several studies have implicated the differentiation status of therapeutic cells to affect their longevity, expansion, and anti-tumor efficacy [137–140]. Based on human viral infection studies, the various modalities for differentiation status (stem cell memory, central memory, and effector memory) can be distinguished using surface markers (CD45RA, CD45RO, CD62L, CCR7, CD27, and CD28) [131, 138]. Where naive/stem cell memory T cells consist of (CCR7^+^, CD62L^+^, CD45RA^+^, CD27^+^, CD28^+^, and CD45RO^−^ markers) and progress to effector memory/exhausted T cells (CCR7^−^, CD62L^−^, CD45RA^±^, CD27^−^, CD28^−^, and CD45RO^±^ [141, 142]. Correlating the differentiation status of therapeutic cells to tumor killing efficacy have shown the propensity for naive and early memory phenotypes to have enhanced tumor eradicating properties compared to more differentiated phenotypes [137, 141, 143]. Fraietta et al. revealed that the extent of sustained remission within responding CLL patients to CD19 CAR-T cells, correlated with increased frequency of memory markers CD27^+^CD45RO^−^CD8^+^ T cells prior to CAR-T cell generation [137]. Additionally, Powell et al. attributed stable numbers of CD27^+^CD28^+^ tumor reactive T cells to contribute to the development of long-term, melanoma-reactive memory CD8^+^ T cells and eradication of melanoma tumors in patients [141]. Within a phase 1 clinical trial for relapsed B cell malignancies where of the 16 patients treated with CD19/CD20 bispecific CAR-T cells, clinical responders (CR or PR) had increased naive and central memory T cells in apheresis product compared to non-responders [141]. Although final CAR-T cell products for both responders and non-responders were primary effector memory phenotypes, the differentiation status of the starting material potential impact the efficacy [141]. These results establish that the differentiation status for both the starting material and final CAR-T cell products offers a biomarker to potentially predict long term persistence and subsequently anti-tumor efficacy in patients. In addition to their use for biomarkers, the differentiation state of the therapeutic cell is also being considered as efforts are ongoing to produce less differentiated and naïve therapeutic cells with enhanced efficacy [139, 144].

### Cytokine release syndrome (CRS) biomarkers

CRS is a common side effect often arising from cell-based therapies and is a prevalent problem that restricts its application [145, 146]. Following CAR-T cell activation, therapeutic cells and endogenous cell types (monocytes, endothelial and stromal cells) become activated and release proinflammatory cytokines (IL-6 and TNF-α) causing symptoms including high fever, organ failure, and death [145, 146]. Therefore, identifying biomarkers to predict early onset of CRS induced by cell-based therapies can expand its application and result in a safer treatment strategy. Within a CD19 CAR-T cell phase 1 dose-escalation trial for ALL patients, significantly higher levels of C-reactive protein (CRP) were seen in patients who had severe CRS compared to those with mild or no CRS [147]. CRP is produced by the liver in response to inflammation and particularly IL-6. Additional associations between CRS development and concentrations of IL-6, IL-10, interferon-γ, and GM-CSF, were also observed [147]. Hay et al. observed higher levels of von willebrand factor (VWF), a molecule secreted by endothelial cells upon activation, in CD19-targeting CAR-T cells treated patients’ serum cells with grade 4 ≥ CRS compared to patients with grade ≤ 3 CRS [148]. An increase in angiopoietin-2 and a decrease in angiopoietin-1 in patients’ serum was associated with heightened severity of CRS as well [148]. Analyzing 133 patients treated with CD19/CD22 CAR-T cells, Sheth et al. developed a model to identify patients with grade ≥ 4 CRS as developing a fever ≥ 38.9 °C within CAR-T cell administration in conjunction with serum MCP-1 concentration ≥ 1343.5 pg/mL [145]. Other modalities in which to predict CRS include serum levels of ferritin, LDH, aminotransferase (AST), alanine aminotransferase (ALT), blood urea nitrogen (BUN), and creatinine [131]. The identification of predictive biomarkers for CRS associated with cell-based therapies is crucial to enhance their safety and broaden their therapeutic potential.

### TME cellular composition biomarkers

The discovery of biomarkers furthering our understanding of CAR-T cell therapy paves the way for optimization of safer and more efficacious cellular therapies. Biomarker linked to the composition of the TME offer an additional method to predict cell-based outcomes. An increase in effector immune cells within the TME has been linked to enhanced CAR-T cell infiltration and efficacy [112, 132] and a decrease in monocytic MDSC counts (CD14^+^CD33^+^HLA-DR^+^ cells) has been associated with better responses to CD19-targeting CAR-T therapy for patients with B cell malignancies [131, 149]. Therefore, profiling the cellular composition of the TME can provide a biomarker to anticipate and monitor the efficacy of CAR-T cell therapy. Future studies to identify biomarkers to detect, monitor, and predict mechanisms of immune evasion can further the development of more personalized treatment regimens that will overall enhance the efficacy of cellular therapies.

## Conclusion

Cell-based therapies, such as CAR-T cell therapy, have shown remarkable effectiveness in combatting hematological malignancies but have encountered substantial challenges when applied to the treatment of solid tumors. The limitations in treating solid tumors can be primarily attributed to the pervasive immune evasion mechanisms present within the TME. In this comprehensive review, we aim to provide an overview of the diverse mechanisms employed by tumors to evade immune responses and present innovative strategies to surmount these hurdles.

The elucidation of these immune evasion mechanisms has paved the way for the development of cellular products engineered to overcome and withstand the immunosuppressive influences within the TME. These advancements have significantly bolstered the efficacy of cell-based therapies in confronting solid tumors. To further enhance our capabilities in this field, a deeper understanding of the intricacies of immune evasion, coupled with ongoing advancements in cellular engineering, is paramount.

Furthermore, the establishment of novel biomarkers has greatly augmented our comprehension of cell-based therapies. These biomarkers hold the potential to not only enhance safety measures but also facilitate the tailoring of treatment strategies to individual patients, ultimately improving overall therapeutic efficacy. The utilization of cell-based therapeutics for the treatment of solid tumors represents a promising avenue that necessitates a comprehensive exploration of immune evasion tactics within the TME, continuous innovation in cellular engineering techniques, and the identification of predictive biomarkers. This collective effort may yield more potent and effective cellular therapies for solid tumor treatment.

## Data Availability

Not applicable.

## Abbreviations

CBI: Cell-based immunotherapy
CAR: Chimeric antigen receptor
TME: Tumor microenvironment
CAR-T: CAR-engineered T
CAR-NK: CAR-engineered NK
ALL: Acute lymphoblastic leukemia
MM: Multiple myeloma
AML: Acute myeloid leukemia
BCMA: B cell maturation antigen
MHC: Major histocompatibility complex
CTLA-4: Cytotoxic T lymphocyte-associated molecule-4
PD-1: Programmed cell death receptor-1
PD-L1: Programmed cell death ligand-1
TIM-3: T cell immunoglobulin mucin-3
LAG-3: Lymphocyte-activation gene 3
MDSC: Myeloid-derived suppressor cell
TIL: Tumor infiltrating leukocyte
TAM: Tumor-associated macrophage
TGF-β: Transforming growth factor-beta
CSC: Cancer stem cell
TAA: Tumor associated antigen
TanCAR: Tandem CAR
iNKT: Invariant natural killer T
MAIT: Mucosal associated invariant T
γδ T: Gamma delta T
NKR: Natural killer receptor
ECM: Extracellular matrix
EGFR: Epidermal growth factor receptor
MPM: Malignant pleural mesothelioma
CPB: Checkpoint blockade
PR: Partial response
CR: Complete response
CRS: Cytokine release syndrome
VWF: Von willebrand factor
